# Curcumin modulates cell death and is protective in Huntington’s disease model

**DOI:** 10.1038/srep18736

**Published:** 2016-01-05

**Authors:** Anjalika Chongtham, Namita Agrawal

**Affiliations:** 1 Department of Zoology, University of Delhi, Delhi, India-110007

## Abstract

Huntington’s disease (HD) is a progressive, dominantly inherited neurological disorder caused by an abnormal expansion of polyglutamine (polyQ) repeat within the Huntingtin (Htt) protein with no disease modifying treatments. In a *Drosophila* model of HD, expression of mutant Huntingtin (Htt) protein with expanded polyQ leads to formation of inclusion bodies (IBs), increase in cellular toxicity, progression of motor disabilities and reduced viability. Multiple cellular events such as oxidative stress, mitochondrial dysfunction, inflammation and transcriptional dysregulation are reported to contribute to pathology, however, till date there are no disease-modifying treatments with least side effects. Therefore, we investigated effect of the phytochemical curcumin on HD pathogenesis. Curcumin, a phytochemical and commonly used ingredient in Asian food has a wide spectrum of anti-oxidant, anti-inflammatory and anti-fibrilogenic properties. In this study, we provide evidence that curcumin significantly ameliorates disease symptoms in a *Drosophila* model of HD by suppressing cell death and can be a key to halting the progression of Huntington’s disease with least side effects.

Huntingtin (Htt) is a large 350 kDa protein which is expressed ubiquitously and normally carries a repeat of ~8–25 glutamines in its N-terminal portion^1^. Huntington’s disease (HD) is a dominant neurodegenerative disorder caused by an expansion of CAG tract beyond 35 repeats in exon 1 of the IT15 gene encoding Htt protein. Increasing evidences suggest that most of the neurodegenerative diseases share important pathogenic symptoms even though the clinical features and pattern of neurodegeneration are different. The signature profile of neurodegenerative diseases is that they are late-onset, progressive and ultimately fatal disorders which manifest as movement disorders, cognitive deficits and neuropsychiatric disturbances. Disease is characterized by several molecular and cellular abnormalities, however, a pivotal set of studies in diseased mouse and human patients suggest cell death to be one of the most consistent neuropathological abnormalities in Huntington’s disease. The characteristic disease symptoms result from selective death and dysfunction of specific subsets of neurons within the central nervous system^2^,^3^.

Currently, there is no cure or effective therapy for HD or other neurodegenerative diseases, but pivotal studies suggest tight correlation between cell death and neurodegenerative diseases. Therefore, identification of new therapeutic strategies aimed to modulate cell death for preventing or suppressing disease progression may define the desired outcome. One of the major drawbacks of currently available treatments for polyQ diseases is that they result in multiple side effects and only a few of them can alleviate disease symptoms. Therefore, we focused on testing a phytochemical ‘curcumin’ that could counteract multiple pathways specially cell death involved in Huntington’s disease without any serious side effects.

A comprehensive understanding of the molecular aspects and pathophysiology of polyQ diseases is necessary for finding potential therapeutic drugs or treatments which can slow down disease progression or delay disease onset. Although the mechanisms through which aggregation-prone mutant proteins cause neurodegeneration remain unresolved, there are well-established links demonstrating their roles in dysregulation of multiple cellular processes and functions such as transcription, cell signaling, mitochondrial function, oxidative stress, inflammation and apoptosis^4^,^5^,^6^,^7^. In this scenario, administration of plant-derived compounds with multiple cellular targets is anticipated to attain greater therapeutic efficacy as opposed to rationally-designed mono-targeted agents in the treatment of multi-faceted polyQ diseases.

Curcumin ((1E, 6E)-1, 7-bis (4-hydroxy-3-methoxyphenyl)-1, 6-heptadiene-3, 5-dione), a polyphenolic compound with an outstanding safety profile is the major bioactive component of turmeric, a spice commonly used in Indian and Asian food and medicine. Multiple therapeutic beneficial effects of curcumin, which could be linked to its ability to act as a strong antioxidant, anti-inflammatory phytochemical and anti-protein aggregation effect have been reported during the last 10 years. It has been extensively used in the treatment of several medical conditions like cystic fibrosis, cancer, gastric ulcers, liver diseases and arthritis. Curcumin is also reported as a preventive measure for prevention and treatment of Alzheimer’s disease (AD)^8^,^9^,^10^. Several other studies have also demonstrated that curcumin has strong neuroprotective antioxidant properties; for instance, scavenging ROS^11^ and neutralizing nitric oxide (NO-) based free radicals^12^.

In this study, we used *Drosophila* model of polyQ diseases to test the efficacy of curcumin in alleviating disease symptoms. Transgenic *Drosophila* has proven to be an excellent model system to study neurodegenerative diseases because it replicates most of the features of the disease condition, for instance, late-onset, decreased lifespan, neurodegeneration, motor dysfunction and progressive accumulation of aggregates in cytoplasm and neurites^4^,^13^,^14^,^15^.The fly model has been useful to address whether the diverse, yet overlapping, pathogenic features of polyQ diseases are due to altered activities of disease-specific protein or to an intrinsic cytotoxic effect of expanded polyQ chains themselves.

The exon1 fragment of mutant Htt with 93 glutamine residues (Httex1p Q93) as well as expanded polyQ peptides alone are intrinsically cytotoxic and cause early adult death and neurodegeneration in *Drosophila*^16^,^17^. These studies show that a large part of pathology is due to dominant cytotoxic effects of expanded repeat polyQs themselves. Therefore, the pathogenic mechanisms of polyQ diseases may share similar cellular and biochemical characteristics that allow therapeutic strategy for one disease to be effective in the others.

Here, we show that curcumin suppresses degeneration of photoreceptor neurons, indicative of disrupted internal eye architecture, in transgenic *Drosophila* expressing Httex1p Q93 and Q48 peptides. We also found that curcumin ameliorates extensive degeneration and external dysmorphology of eye caused by expression of Httex1p Q93 in all cells of the *Drosophila* eye. Our results indicate that in addition to reducing neuronal loss, curcumin improved polyQ induced motor neuronal dysfunction. Moreover, dietary curcumin did not affect the integrity of photoreceptor cells of adult eye and motor neuron function of transgenic *Drosophila* expressing the first exon of Htt with a normal-range repeat of 20 glutamine residues (Httex1p Q20).

Since apoptosis has been linked to neuronal death in polyQ diseases including HD^3^,^18^,^19^, we investigated if curcumin can protect against polyQ-mediated cell death in eye discs. We found less prevalence of apoptotic cell death in third-instar larval eye discs attributable to curcumin administration. Our studies suggest that curcumin may be a potential therapeutic drug for HD and other polyQ diseases as it may harbor inherent ability of suppressing polyQ-induced neurodegeneration with no adverse side effects, when administered at disease onset or even after the disease has progressed.

## Materials and Methods

### ***Drosophila*** stocks and dietary condition

The polyglutamine expressing transgenic lines used in the present study were w; P {UAS-Httex1p Q20}, w [*] P {w [+mC] = UAS-Q48.myc/flag} and w; P {UAS-Httex1p Q93}4F1^17^,^20^. The GAL4 drivers used in the experiments were the pan-neuronal elav driver w; P {w^+mW.hs^ = GawB} elavC155 and eye-specific gmr driver w; P [w + mC = GAL4-ninaE.GMR] 12. Appropriate crosses were carried out in order to obtain desired genotypes. Curcumin (Sigma Aldrich) was mixed in fly food at desired concentration (3 μM, 5 μM, 10 μM, 15 μM or 20μM) for growing experimental larvae or flies while controls were reared in normal food devoid of drug at 25 °C. DMSO (Sigma Aldrich) was supplemented in equal amounts in all the food conditions.

### Feeding Assay

Yeast paste with blue colour (FD & C Blue Dye No. 1, Sigma Aldrich) and curcumin at desired concentration (as above) was placed centrally on a petri dish (100 mm × 10 mm) plated with 3.3% (wt/vol) agar. Two groups of 10 feeding early third-instar larvae for each condition were transferred to blue yeast paste and allowed to feed for 2 h. After feeding, each group of larvae were washed in distilled water and dried. Larvae were then homogenised in PBS, centrifuged at 35,000 rpm for 10 min and absorbance at 625 nm was read.

### Crawling Assay

The apparatus was a petri dish of 100 mm × 10 mm dimensions composed of 3.3% agar. A track of 2 mm wide, 30 mm long and 5 mm deep was created in the petri dish. The distance covered in 30 s by each wandering third-instar larva placed in the track was recorded. A total of 10 larvae per condition were monitored for two trials.

### Climbing Assay

The locomotor ability of flies was monitored by their ability to climb up a vertical glass tube (diameter, 2.2 cm). A group of 10 flies were gently tapped to the bottom of the tube. The number of flies that could climb a height of 10 cm within 15 s was recorded. For each condition, 2 groups of 10 flies each were tested in the marked tube for a total of 6 trials.

### Pseudopupil Analysis

This assay allows rapid visualization of rhabdomere arrangement in the ommatidia of the compound eye which is a direct measurement of the number of surviving photoreceptor neurons. The decapitated head of adult fly was mounted on a microscope slide and the eyes were analyzed under Nikon Eclipse (Ni-E) microscope with 50X oil objective. A total of 6 eyes, a minimum of 200 ommatidia were scored per condition and the number of visible rhabdomeres was counted for each ommatidium.

### Rough eye phenotype assessment

Flies expressing Httex1p Q93 driven by *gmr*-GAL4 were grown at 25 °C in normal food and food containing 5 or 10 μM curcumin. The external surface of fly eye was examined at days 1, 7 and 14 post-eclosion with a Nikon SMZ745T microscope, and a total of 30 flies were analyzed for each condition. Photographs of at least 5 fly eyes per condition were taken using a Nikon SMZ1500 microscope.

### Dead cell detection

Acridine orange (AO) staining was performed to observe apoptotic cells in the eye imaginal discs of third-instar larvae. Larvae were dissected in PBS and eye discs were incubated in 5 μg/ml acridine orange (Himedia) solution for 3 min followed by rinsing in PBS. At least 5 discs for each condition were then photographed immediately using a Nikon Eclipse (Ni-E) fluorescent microscope and analyzed with NIS-elements AR software.

### Statistics

Statistics were performed in IBM SPSS statistical package (version 22). Normality of data was assessed by Shapiro-Wilk tests and equal variance using Levene’s test. For normal distribution, statistical analysis was performed using one-way analysis of variance (ANOVA) followed by Tukey’s post hoc test. If data were not normally distributed, a Kruskal-Wallis test was performed followed by Mann-Whitney U test using Bonferroni correction to adjust the probability.

## Results

### Evaluation of drug effects on feeding behaviour

**Figure 1:**
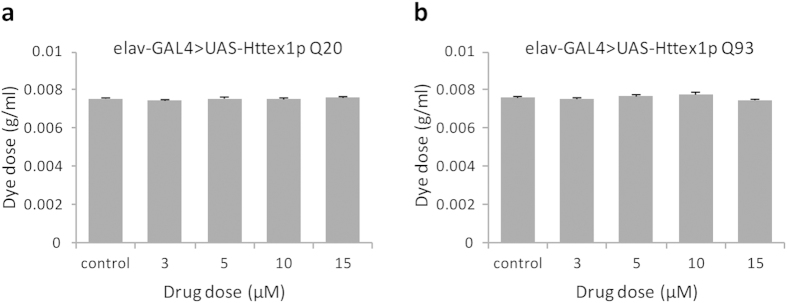
Curcumin has no effect on food intake. Various doses of curcumin were administered to feeding third-instar larvae. No differential feeding behaviour was observed in normal (**a**) Httex1p Q20 as well as diseased (**b**) Httex1p Q93 larvae. For each condition, two groups of 10 larvae were analysed (n = 20) for a total of 6 spectrophotometer readings. Data was analyzed by an analysis of variance (ANOVA) and values represent mean ± SEM.

The vital sense of taste allows animals to prefer palatable and avoid non-nutritious or toxic food. In an attempt to establish a foundation for drug feeding experiments, subsequent interpretation and analysis of results, we monitored larval feeding behaviour. We allowed feeding larvae 2-hour accessibility to a blue-dyed assay plate supplemented with curcumin at various doses and the amount of food consumed was then estimated spectrophotometrically. We found that all concentrations of curcumin including the higher ones had no effect on food intake of larvae expressing normal-length Httex1p Q20 (Fig. 1a) and expanded repeat Httex1p Q93 (Fig. 1b) peptides under the control of *elav*-GAL4 driver. In other words, normal as well as diseased larvae fed curcumin at different doses did not show differential feeding behaviour.

### Curcumin suppresses polyQ-mediated photoreceptor neuron degeneration and internal eye dysmorphology

Neurodegeneration is most readily observed in the fly compound eye composed of approximately 750 repeating units, called ommatidia, each containing eight photoreceptor neurons. These photoreceptor cells produce a stack of tightly packed microvilli that form a photosensitive structure called the rhabdomere. A popular assay of neurodegeneration, commonly called the pseudopupil method, involves counting the photoreceptor neurons of the fly compound eye. These photoreceptor neurons produce a repeating trapezoidal arrangement of 7 visible rhabdomeres in each ommatidium^13^. Progressive loss of photoreceptor cells that can be observed with the pseudopupil technique is seen in flies expressing Httex1p Q93 and Q48 peptides^17^. The pseudopupil assay thus provides a useful quantitative measure of neurodegeneration^13^,^14^.

**Figure 2:**
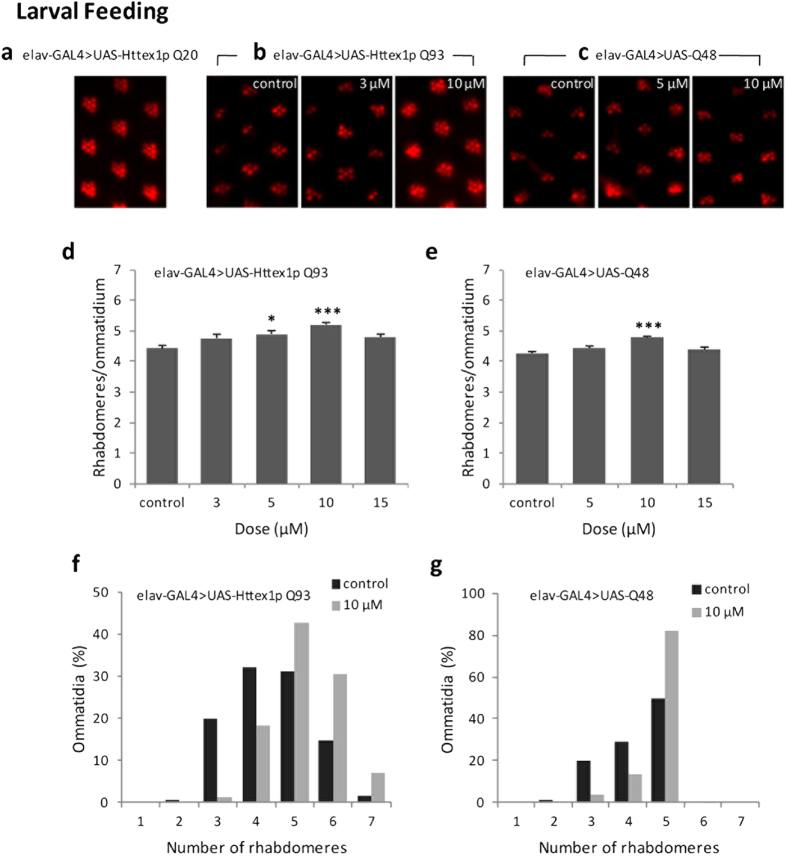
Curcumin administered early during development suppresses photoreceptor neuronal loss. (**a**) Feeding curcumin since larval stage showed no detectable effect in 7-day-old flies expressing Httex1p Q20. Curcumin dose-dependently suppressed photoreceptor neuron degeneration, when administered since larval stage, in Httex1p Q93 (**b,d,f**) and Q48 flies (**c**, **e**, **g**) at 7 day post eclosion. For each condition, at least 200 ommatidia in 6 flies were assayed. Data was quantified as the number of rhabdomeres per ommatidium (**d,e**) and the distribution of the percent of ommatidia (**f,g**) at 7 day post eclosion. Significance was calculated by using an analysis of variance (ANOVA) followed by Tukey’s post hoc test; values represent mean ± SEM (***p < 0.001; *p < 0.05, compared with control).

**Figure 3:**
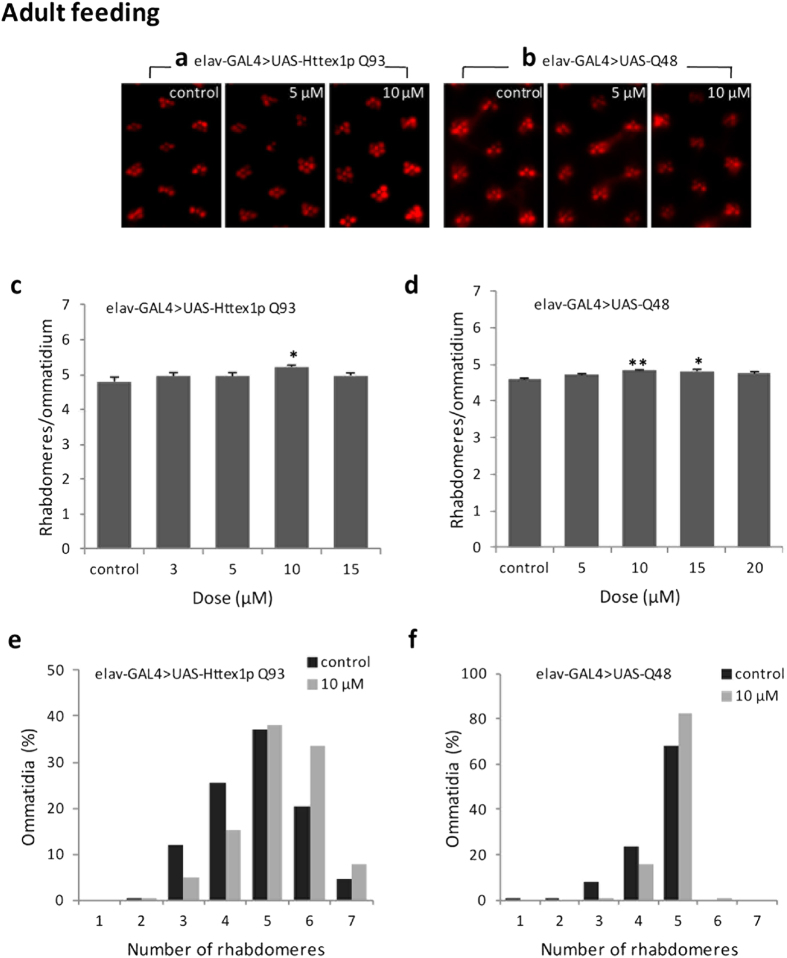
Curcumin administration after disease progression suppresses photoreceptor neurodegeneration. Curcumin administration only during adult stage suppressed photoreceptor neurodegeneration in pathogenic Httex1p Q93 (**a,c,e**) and Q48 flies (**b,d,f**) quantified at 7 day post eclosion. At least 200 ommatidia in 6 flies were examined per condition. Data was quantified as the number of rhabdomeres per ommatidium (**c,d**) and the distribution of the percent of ommatidia (**e,f**). Data analysis was performed using an analysis of variance (ANOVA) followed by Tukey’s post hoc test; values represent mean ± SEM (**p < 0.01; *p < 0.05, compared with control).

We performed the pseudopupil technique at day 7 post-eclosion to study the effects of curcumin on the integrity of photoreceptor neurons and internal morphology of eye in flies expressing normal-range repeat Httex1p Q20, expanded repeat Httex1p Q93 and expanded polyglutamine peptides (Q48) under the control of *elav*-GAL4. Httex1p Q20 flies grown in normal or curcumin-supplemented food exhibited the seven characteristically arranged rhabdomeres in each ommatidial unit irrespective of different doses (5, 10 and 15 μM doses) of the drug administered (Fig. 2a). Rearing Httex1p Q93 and Q48 flies on curcumin-supplemented food from first larval instar (Fig. 2b–g) or only during adult period (Fig. 3a–f) suppressed photoreceptor neuron degeneration in a dose-dependent manner. The most significant suppression was achieved at 10 μM dose fed either at the time of disease onset (larval stage) or even at a later stage when the disease has progressed (adult period). This finding clearly demonstrates that dietary curcumin suppresses polyQ-induced neurodegeneration and internal eye morphological defects with no side effects.

### Curcumin mediates attenuation of polyQ-induced rough eye phenotype and pigment loss indicative of cytotoxicity

*Drosophila* eye development provides a sensitive assay for studying the effects of polyQ expansion on cell differentiation and viability of both neuronal and non-neuronal cells. The cells that are predestined to form the eye grow during the larval period as uncommitted cell types. A morphogenetic wave passes across the eye during the third-instar stage and as the wave passes, cells become committed to particular fates in the eye^21^ which include the neuronal cells (photoreceptors and mechanoreceptors) as well as pigment cells.

The *elav* and *gmr*-GAL4 are two widely used drivers for neurodegeneration studies. The *elav* promoter expresses GAL4 in all cells of the nervous system beginning from embryogenesis and continues into adult life^22^. On the other hand, the *gmr* promoter is expressed in the differentiating photoreceptor neurons and pigment cells in the third instar eye imaginal disc^23^. Expression of polyQ containing proteins by *elav* promoter can lead to neurodegeneration but it does not affect external morphology. However, *gmr*-mediated expression of the mutant proteins causes massive degeneration of the eye and overt external dysmorphology^16^,^24^,^25^,^26^.

**Figure 4:**
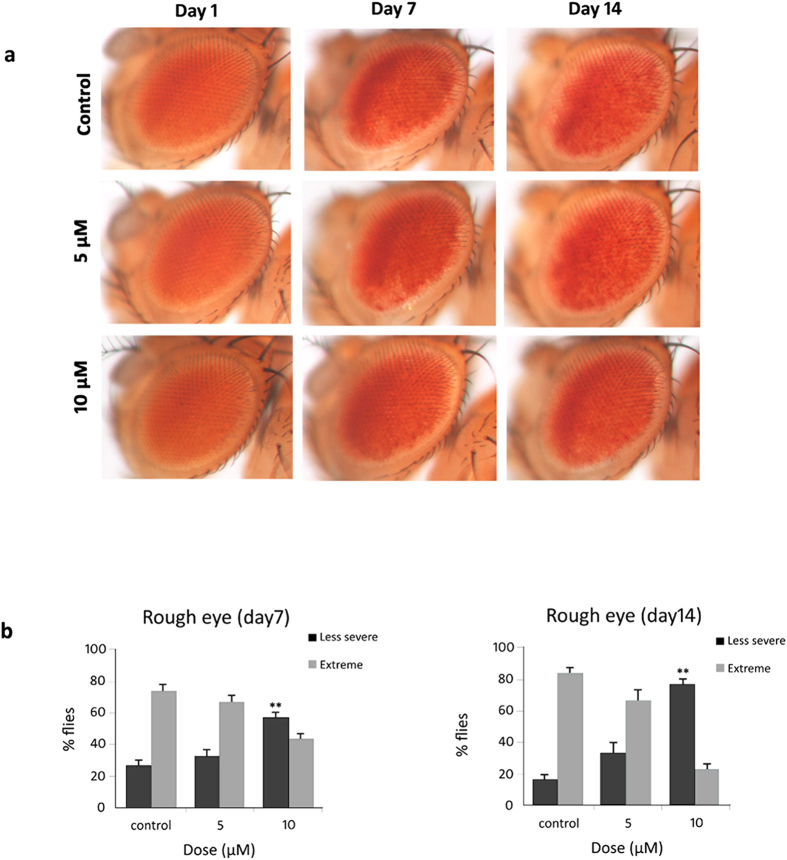
Dietary supplement of curcumin suppresses polyQ-induced rough eye phenotype and loss of pigmentation. (**a**) Images of external eye surface of 1-day, 7-day and 14-day old flies expressing Httex1p Q93 driven by *gmr*-GAL4 reared on normal food (*top panels*), 5 μM (*middle panels*) and 10 μM (*bottom panels*) curcumin-containing food. Dietary curcumin mediated attenuation of polyQ-induced degeneration and pigment loss indicative of cytotoxicity is evident at days 7 and 14 post-eclosion. (**b**) Administration of 10 μM curcumin suppressed rough eye phenotype and pigment loss at days 7 (*left panel*) and 14 (*right panel*). The frequency of necrotic lesions in the fly eye was evaluated by considering less severe phenotype as 0–4 necrotic lesions and extreme phenotype as >4 necrotic lesions. Data represents the mean ± SEM of values obtained from 30 flies per condition (n = 30); **p < 0.01 compared with control. Data was analyzed using the Kruskal-Wallis test followed by Mann-Whitney test with Bonferroni correction of *p*-values.

To monitor external effect on eye, we used *gmr*-GAL4 driver to express Httex1p Q93 transgene in the fly compound eye. Our results clearly indicate that administration of 10 μM curcumin from early larval stage can mitigate Httex1p Q93 induced cytotoxicity characterised by visible external morphological defects such as rough eye phenotype with necrotic lesions and loss of pigmentation (Fig. 4a,b). The eyes of 1-day-old flies appeared normal with only slight roughness and pigment loss. However, with aging, eye pigmentation decreases with increase in roughness and necrotic lesions, thereby suggesting progressive degeneration of pigment cells. Nevertheless, this defective eye phenotype was significantly suppressed by 10 μM dose of curcumin at days 7 and 14 post eclosion.

### Curcumin ameliorates polyQ-mediated locomotor dysfunction

Although progressive and overt neuronal loss is a key hallmark of HD and other polyQ diseases, deficits can also be due to compromised neuronal functioning. As common measures of neuronal dysfunction, we examined the loss of larval crawling and adult climbing ability when transgenes are expressed in neurons.

Expression of the first exon of human Htt with normal-repeat-length of 20Qs (Httex1p Q20) has no toxic effect on its own^27^. However, flies which express expanded repeat Httex1p Q93 or expanded polyQ peptide alone exhibit progressive abnormal movements with age because of impaired motor neuronal function^13^,^14^. We evaluated the effects of curcumin on motor function of flies expressing Httex1p Q20, Httex1p Q93 or expanded polyQ peptide alone (Q48) under the control of *elav*-GAL4 driver.

**Figure 5:**
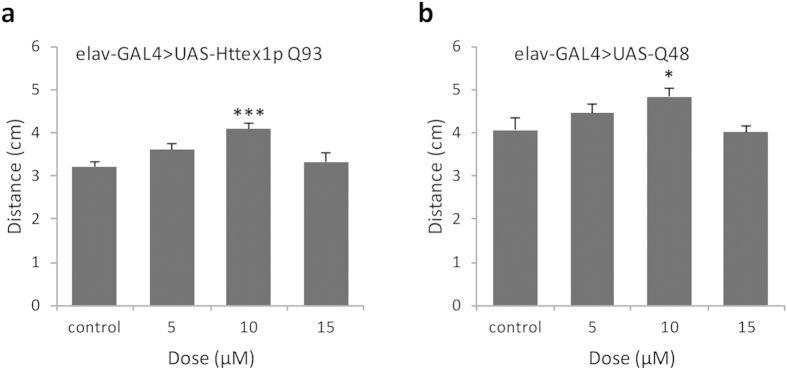
Curcumin improves impaired locomotor activity of polyQ larvae. (**a**,**b**) The crawling ability of 3^rd^ instar larvae at different doses was evaluated. Impaired motor function was suppressed at 10 μM in larvae expressing Httex1p Q93 (**a**) or Q48 (**b**). For each condition, the average of 10 larvae was calculated for two trials (n = 10). Data analysis was performed using an analysis of variance (ANOVA) followed by Tukey’s post hoc test; values represent mean ± SEM (***p < 0.001; *p < 0.05, compared with control).

To quantify locomotor dysfunction, Httex1p Q93 and Q48 larvae were fed increasing concentrations of curcumin and a simple measure of locomotor function called the ‘crawling assay’ was performed. We found that feeding curcumin improved the crawling ability of both Httex1p Q93 and Q48 larvae, in a concentration-dependent manner. Marked improvement in larval crawling behaviour was observed at 10 μM dose of curcumin (Fig. 5a,b).

**Figure 6:**
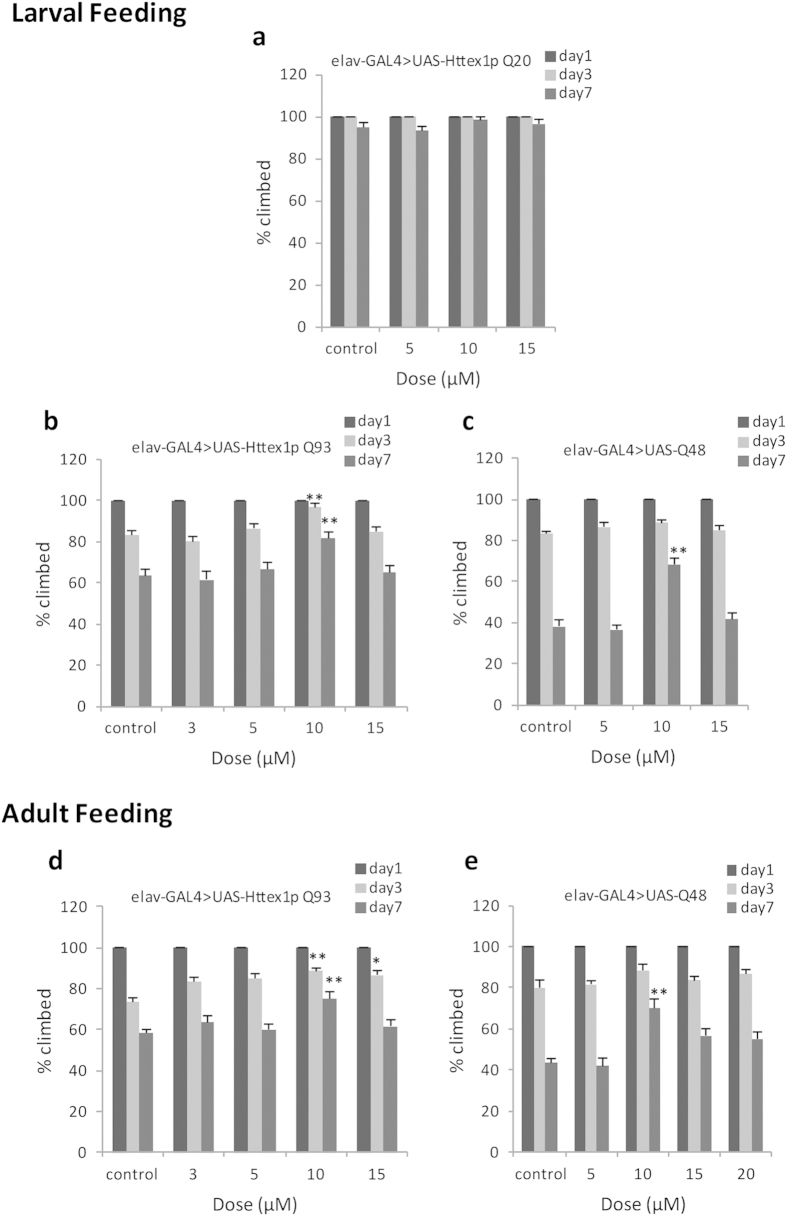
Curcumin improves locomotor dysfunction of polyQ flies. Climbing ability of adults aged day 1, 3 and 7 post eclosion, fed normal or curcumin-containing food was evaluated. (**a**) All doses of curcumin fed since larval stage had no detectable effect on locomotor activity of non pathogenic Httex1p Q20 flies. Administration of 10 μM curcumin since larval stage (**b,c**) or only during adult period (**d,e**) suppressed motor dysfunction in flies expressing Httex1p Q93 and Q48. Comparison was made between control and drug treated flies of same age. For each condition, the climbing ability of two groups of 10 flies were monitored (n = 20) for a total of 6 trials. Analysis of data was done using the Kruskal-Wallis test followed by Mann-Whitney test with Bonferroni correction to adjust *p*-values; data represents mean ± SEM (**p < 0.01; *p < 0.05, compared with control).

For addressing motor function of adults, the climbing ability of flies grown on food supplemented with different doses of curcumin was evaluated at days 1, 3 and 7 post-eclosion. Curcumin showed no detectable effect on motor ability of Httex1p Q20 flies reared on curcumin-containing food since larval stage (Fig. 6a). On the contrary, we found that motor dysfunction of Httex1p Q93 as well as Q48 flies were progressive and most prominent on day 7 post-eclosion. Furthermore, our results indicate that feeding curcumin, from the beginning of larval life (Fig. 6b,c) or only after emerging from the pupal case as adults (Fig. 6d,e) suppressed the progressive loss of motor function, in a dose-dependent manner.

### Curcumin reduces polyQ-induced cytotoxicity and cell death

Apoptosis, or programmed cell death, is an intrinsic and highly organized form of cell death which is common in various biological processes and pathological conditions. Aberrant apoptosis in the central nervous system has been implicated in the pathogenesis of a broad array of neurological disorders, including polyQ diseases^19^,^28^,^29^. Earlier reports suggest that mutant Htt induces neurodegeneration by an apoptotic mechanism and that specific inhibitors of apoptosis avert mutant Htt-induced neuronal death^3^,^19^. The N-terminal Htt fragments with expanded repeat length are prone to misfolding and aggregation and appear to cause cytotoxicity and severe neurological phenotypes^30^,^31^. Several studies demonstrate that misfolded proteins affect normal mitochondrial function and alter intracellular signalling thereby eliciting apoptosis^19^,^28^,^29^.

**Figure 7:**
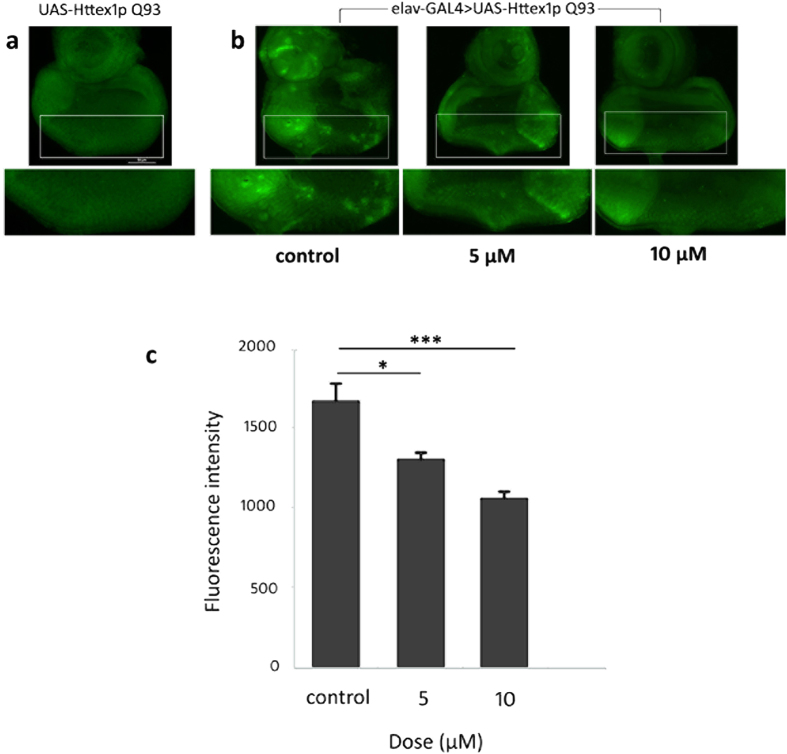
Curcumin suppresses polyQ-mediated cytotoxicity and cell death. Acridine orange (AO) staining of third-instar eye discs from (**a**) UAS-Httex1p Q93 larvae (non pathogenic) showing negligible cell death and (**b**) elav-GAL4 > UAS-Httex1p Q93 larvae (pathogenic) with varying levels of cell death. Rearing on 5 μM curcumin partially reduced cell death while growing on 10 μM curcumin significantly reduced the abundance of AO-positive dead cells. Histogram in (**c**) represents the mean fluorescence intensity (measured in arbitrary fluorescence units) of acridine orange staining in elav-GAL4 > UAS-Httex1p Q93 (pathogenic) eye discs of larvae grown on different curcumin concentrations. Data represents mean ± SEM of values obtained in 5 larvae per condition (n = 5). Statistical significance: ***p < 0.001; *p < 0.05, compared with control. Analysis of data was performed using an analysis of variance (ANOVA) followed by Tukey’s post hoc test.

Curcumin, a potent antioxidant compound, has been found earlier to alleviate cytotoxicity, oxidative stress and apoptotic cell death in neurodegenerative diseases^32^,^33^,^34^,^35^,^36^. In an attempt to unravel the mechanism by which curcumin suppresses neurodegeneration and neuronal dysfunction, we performed acridine orange (AO) staining of third-instar eye discs expressing UAS-Httex1p Q93 alone (Fig. 7a) and *elav*-GAL4-driven Httex1p Q93 eye discs from third-instar larvae fed normal or curcumin-supplemented food (Fig. 7b) to identify apoptotic cells. This revealed a slight and considerable reduction in apoptotic cell death in eye discs from larvae fed 5 and 10 μM curcumin respectively in comparison to control. Comparison of the fluorescence intensity of eye discs stained with AO further confirmed that feeding curcumin-supplemented food significantly suppressed the abundance of AO positive cells (Fig. 7c). Therefore, an effective concentration of curcumin ameliorates polyQ-induced cytotoxicity by protecting cells against undergoing apoptosis.

## Discussion

The polyQ diseases manifest as selective neuronal cell death leading to distinct, overlapping, clinical and pathogenic attributes^37^. From a therapeutic perspective, it is encouraging that polyQ diseases may share toxic or disrupted fundamental cellular events because therapy for one disease may be effective in others. Unfortunately, current therapeutic strategies against polyQ diseases including HD fail to have neuroprotective effects or the potential to alter the course of disease. In addition, novel synthetic drugs are highly toxic and elicit severe side effects^38^. Interestingly, the use of phytochemicals in the treatment of neurodegenerative diseases as an alternative to target-specific, synthetic drugs comes with several advantages, such as minimum or no side effects, and low toxicity to normal cells. Natural products that target multiple cellular events are anticipated to play a key role in developing new therapeutic leads for polyQ diseases.

The prevalence of neurodegenerative diseases among people living in the Asian subcontinent, where people regularly consume spices, is lower than in western countries. Research over the last 10 years has shown that phytochemicals derived from several spices target inflammatory pathways and may be effective in treating neurodegenerative diseases^39^. Due to its broad spectrum of therapeutic properties (e.g., anti-oxidant and anti-inflammatory activities) and excellent safety profile, “curcumin”, the most active component in turmeric, has great potential for the treatment of multiple neurodegenerative diseases for which current therapeutics are less than optimal. In our present study, we show that curcumin did not pose detrimental effects in flies expressing normal repeat Httex1p Q20 while effectively protects against polyQ-induced cytotoxicity in transgenic *Drosophila* expressing a mutant Htt exon 1 fragment with expanded polyQ peptide (Httex1p Q93) or expanded polyQ peptide (Q48) alone which is free of any disease gene context. Rearing on curcumin-supplemented diet improved different neurodegeneration phenotypes, viz. progressive degeneration in the eye, eye dysmorphology, organisation of rhabdomeres, and impaired motor function in both Httex1p Q93 and Q48 transgenic *Drosophila*. Since polyQ diseases share common pathogenic features, curcumin is a promising agent for the prevention or treatment of polyQ disorders including HD. We demonstrate that dietary curcumin administered since larval stage or only during adult period ameliorates compromised functioning of neurons. Our results indicate that curcumin suppresses neurodegeneration when it is being inflicted and markedly retard further neuronal degeneration. Continuous synthesis of expanded polyQ protein and neuronal damage during the non-feeding pupal period may have reduced the extent of recovery in eye morphology and motor function.

The identification of genetic and environmental factors responsible for neurodegenerative diseases has augmented evidences for a common pathway of neuronal death viz. apoptosis that involves oxidative stress, perturbed calcium homeostasis, mitochondrial dysfunction and caspase activation^19^. In an attempt to gain mechanistic insight into the neuroprotective effects of curcumin, we investigated if curcumin attenuates polyQ-induced apoptotic cell death, which underlies the symptoms of many neurodegenerative diseases including HD, in transgenic *Drosophila* expressing expanded Httex1p Q93 protein fragment driven by the pan-neuronal *elav*-GAL4. We found a substantial reduction in polyQ-induced cell death in third-instar eye discs due to administration of curcumin which has been implicated in alleviating cytotoxicity, oxidative stress and apoptotic cell death in neurological disorders^32^,^33^,^34^,^35^,^36^. Therefore, curcumin modulates polyQ-mediated cytotoxicity in fly models by suppressing apoptotic cell death with a consequent improvement in disease symptoms.

## Conclusions

PolyQ pathology is complex and may affect multiple cellular events. Therefore, administration of therapeutic agents with least side effects that target multiple cellular processes and functions are postulated to achieve better relief from pathology than the mono-targeted agents. Phytochemical curcumin is known to cross the blood-brain barrier, has an excellent safety profile, number of pleiotropic properties and therefore, has tremendous potential for neuroprotective efficacy. Our findings strongly suggest that curcumin ameliorates polyQ-induced compromised neuronal function, neurodegeneration, and cytotoxicity and can be a promising candidate for the treatment of polyQ disease patients with least side effects.

## Additional Information

**How to cite this article**: Chongtham, A. and Agrawal, N. Curcumin modulates cell death and is protective in Huntington′s disease model. *Sci. Rep.* **6**, 18736; doi: 10.1038/srep18736 (2016).
